# Visual estimation of the force applied by another person

**DOI:** 10.1038/s41598-022-10243-7

**Published:** 2022-04-13

**Authors:** Yusuke Ujitoko, Takahiro Kawabe

**Affiliations:** NTT Communication Science Laboratories, Nippon Telegraph and Telephone Corporation, Atsugi, 243-0198 Japan

**Keywords:** Psychology, Human behaviour

## Abstract

As observers, we believe that we can visually estimate the force that another person is applying to a material. However, it is unclear what kind of cues we use to do this. We focused on two types of visual change that occur when actors push an elastic material from above with their fingers: visual shaking and visual indentation depth. The first one relates to a finger/hand shaking, known as an “induced tremor”, and the second one relates to material deformation due to the application of force. We found that human observers mainly used visual shaking to estimate the force being applied by another person in a video clip. Overall, the apparent applied force was perceived to be stronger when the level of visual shaking was greater. We also found that observers mainly used visual indentation depth and visual shaking to estimate the softness rating of materials. Overall, the apparent softness was perceived to be greater when the visual indentation depth was larger and the level of visual shaking was lower, which indicates that observers use visual shaking to estimate the force being applied, and that estimated force is then used for an estimation of softness.

## Introduction

By observing the motor actions of another person, we can infer his/her intention, anticipate the consequence of his/her action, and estimate the physical properties of a material that he/she is manipulating^1–8^. For example, professional basketball players can predict the results of another player’s shot by visually reading the shooter’s body kinematics^1^. Interestingly, in Aglioti’s study^1^, basketball players watching a video exhibited time-selective motor activation, consistent with previous evidence for the close relationship between seeing and conducting motor action in the brain^9^. Indeed, the combination of observation and imitation of another person’s action has been found to be effective for a non-athlete group in learning new sports^10^. As a candidate mechanism for the recognition of another person’s action by humans, the mirror neuron system has been investigated^11^. These neurons, originally discovered in the ventral premotor area F5 of the macaque monkey^12^, respond both during action execution and observation, and are thought to constitute a system fundamental for understanding goal-directed actions^13^. The ability of the observer to visually read the kinematics of another person’s action is also useful for judging the intrinsic property of a material. Specifically, the kinematics of another person’s hand motion has been used as a cue to predict the size^5,6^ and weight^7,8^ of a material that he/she is about to grasp.

The ability of human observers to visually determine the amount of force another person is applying to a material has been investigated. Some behavioral studies have already indicated that the weight of a box (which strongly relates to the force required to lift it) can be inferred quite accurately by observing another person lifting it^14,15^. A single-pulse transcranial magnetic stimulation (TMS) study has demonstrated that action observation induced a dynamic simulation of the observed movement in the observer’s motor system^16,17^.

However, previous studies did not pay attention to the phenomenon of induced tremor (also called physiological tremor), which is one of the bodily effects that often occur when a person applies force to a material. An induced tremor is a self-maintaining force fluctuation observed when one’s muscles contract anisometrically under appropriate loading^18^. Induced tremors have been confirmed at various body sites such as fingers^19^, hands^20^, or limbs^18^. A tremor has been induced by a minimum pressing force between thumb and index finger of 0.2 kg^21^. The interaction between an actor’s body and any object such as a flat surface or stick can induce a tremor. Depending on the loading strength, the power peak of an induced tremor is observed at a temporal frequency of 4–8 Hz. The tremor occurs involuntarily and cannot be voluntarily suppressed as a physical response. Hence, it is plausible to assume that human observers naturally use the visual features of an induced tremor to judge the amount of force that another person is applying to an external material. In addition, the visual features of an induced tremor can be imitated even when little force is exerted. We were interested in whether such imitated tremors could lead to an increased perception of force.

In addition to changes in another person’s physical behavior, the amount of change observed in the appearance of a material may also be a cue to the force being applied by that person. For example, the more force a player transfers to a ball when hitting it with a golf club, the farther the ball will fly. The stronger the force with which a cushion is pushed in, the larger the cushion’s deformation is. It has not been clarified whether a differing degree of visual change in the appearance of a material could contribute to the determination of the force applied.

**Figure 1:**
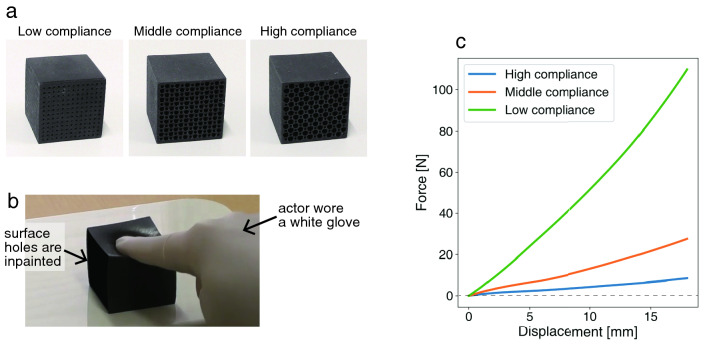
**(a)** Appearance of materials. The material compliance of the three materials was different due to the different sized cylindrical holes inside them. **(b)** Snapshot of a video clip. **(c)** Force–displacement curves of the materials.

The main purpose of the present study is to investigate how observers estimate, from visual features, the force applied to a target material by an actor. We anticipated that force estimation by the observers would be influenced by the following two visual features: visual shaking and visual indentation depth. The visual shaking corresponded to the apparent induced tremors of the actor’s finger. The visual indentation depth corresponded to the apparent amount of material deformation. To investigate this, we conducted a psychophysical experiment. Observers watched a stimulus video clip (see Fig. 1b) in which an actor pressed a 3D printed elastic material (see Fig. 1a) with the actor’s finger. The actor pressed the material with three different levels of force, which would be expected to vary the degree of visual shaking originating from induced tremor. In addition to the visual shaking naturally originating from induced tremor, we asked the actor to slightly vibrate the hand and fingers while pushing the material. We call this vibration of the actor’s hand and fingers “imitated shaking”. As shown in the Results section, the imitated shaking in the actor’s hand and fingers had similar temporal frequency characteristics to that of a naturally-induced tremor. We hypothesized that observers would estimate larger forces being applied by the actor when they saw a greater visual shaking of the hand and fingers. We also used several elastic materials, each having a different force–displacement curve. The variation of applied force and force–displacement curves would make the magnitude of deformation (indentation) vary across the materials in proportion to the force–displacement curves (see Fig. 1c). We hypothesized that observers would estimate larger forces when they observed a greater level of visual shaking and larger visual deformations (or indentations) of the material.

In addition to the force estimation, we were interested in the softness estimation of the target material. It is known that the greater the indentation depth, the softer the material is estimated to be^22–24^. On the other hand, it is an open question about how visual shaking influences the softness estimation. More importantly, it is unclear how the force estimation is related to softness estimation. To address them, we investigated the effect of the visual features on softness estimation and discussed its relation to the force estimation.

The results of the experiment revealed that, in general, visual shaking increased the estimated force rating. We also found that observers used both visual indentation depth and visual shaking to estimate the softness rating of materials. Overall, the apparent softness was judged to be greater when the visual indentation depth was larger and the level of visual shaking was lower. The results are consistent with the interpretation that the observers used the visual shaking to estimate the force being applied, and that estimated force was then used for an estimation of softness.

## Results

### Stimuli analysis

The purpose of this study was to investigate how observers estimated, from visual features, the force applied to a target material by an actor. We expected that the degree of force estimated by the observers would be influenced by the following two visual features: visual shaking and visual indentation depth. We describe each feature below.

#### Visual shaking

**Figure 2:**
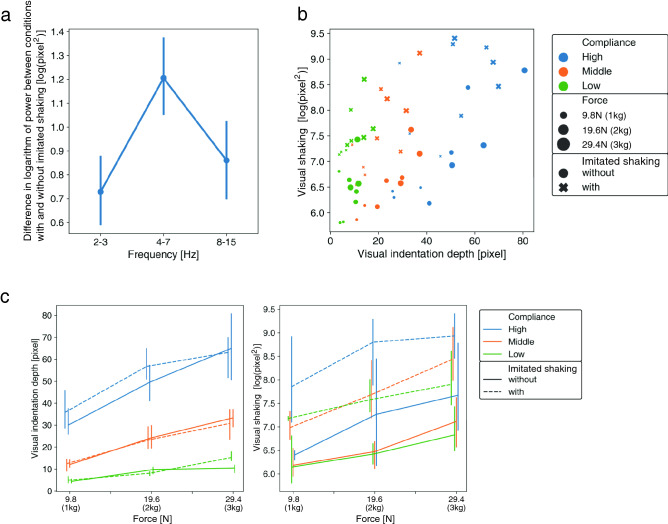
**(a)** Logarithm of power of optical flow vectors. Error bars denote 95%CI. **(b)** Visual features of each video clip. **(c)** Visual feature change for each stimulus factors. Error bars denote 95%CI.

The visual shaking in our experiment corresponded to apparent natural and induced tremors of an actor’s finger and hand shown in a video clip. It was assumed that a larger force caused a larger induced tremor of the finger, which in turn led to a greater level of visual shaking. Therefore, to vary the degree of the visual shaking, we used three force levels. In addition to the visual shaking naturally originating from an induced tremor, we asked actors to slightly vibrate their hand and fingers while pushing the material in order to present various levels of visual shaking. We call the imitated induced tremor “imitated shaking”. Half of the video clips contained the imitated shaking. The other half of the video clips contained only natural induced tremors. To quantify the visual shaking, we analyzed motion vectors at four positions on the actor’s finger: the first joint, the second joint, the third joint, and the fingertip of the index finger. We conducted a fast Fourier transform on the temporal pattern of the motion vectors at each position and calculated the logarithm of power spectrum. As a result, a difference between the conditions with and without imitated shaking was significantly more evident in a specific temporal frequency band (4–7 Hz) that overlapped with temporal frequency bands in which the induced tremor is observed^18^, than in other frequency bands (2–3 Hz or 8–15 Hz) (Fig. 2a). Thus, we define the visual shaking as the logarithm of power spectrum in the 4–7 Hz range at four finger positions. Fig. 2b,c show the variation of the visual shaking depending on the three stimulus factors of the force level, the compliance level, and the imitated shaking condition.

To clarify how the visual shaking changed with the three stimulus factors, we conducted an Aligned Rank Transform (ART)^25^ on the data and then a three-way ANOVA using the three stimulus factors on the aligned ranks. There were significant main effects of force [$df=2$, $F=14.5$, $p<0.001$, $\eta ^{2}_{p}=0.45$], material compliance [$df=2$, $F=8.53$, $p<0.001$, $\eta ^{2}_{p}=0.32$], and imitated shaking [$df=1$, $F=58.46$, $p<0.001$, $\eta ^{2}_{p}=0.62$]. The results of post-hoc test are shown in Supplementary Note 1. The visual shaking increased with the applied force or compliance. Moreover, the level of visual shaking increased when the actors applied imitated shaking. A comparison of effect size shows that the effect of imitated shaking was larger than the effects of other stimulus factors.

We hypothesized that the force rating scores would increase with the visual shaking.

#### Visual indentation depth

The visual indentation depth corresponded to the apparent indentation depth in the videos. To control the visual indentation depth, we configured three levels of force applied by the actor and three levels of material compliance. The actors pushed the top surface of the material with three levels of force, 1 kg (9.8 N), 2 kg (19.6 N), and 3 kg (29.4 N). Each actor wore a white glove to rule out any effect of skin color change, as that can also be a cue to force (see Fig. 1b). We used three materials that had different force–displacement curves (see Fig. 1c). The material contained cylindrical holes whose shape change could be a cue to compliance. To rule out the effect of the holes on the force and softness estimations, we used image editing techniques to inpaint the holes with the color of the surrounding material (see Fig. 1b). We regarded the material’s force–displacement relationships as linear because the R squared values of the fitted linear model that regressed the force with displacement were more than 0.917 (see Supplementary Table 1). We defined the compliance as 0.235 (Low compliance), 0.759 (Middle compliance), and 1.602 (High compliance). As a result, the materials shown in the video clips deformed with various levels of indentation depth and thus generated different levels of perceived visual indentation depth. We hypothesized that the force rating scores would increase with the visual indentation depth. Fig. 2b,c show the variation of the visual indentation depth depending on the three stimulus factors of the force level, the compliance level, and the imitated shaking condition.

To clarify how the visual indentation depth changed with the three stimulus factors just mentioned, we conducted an ART on the data and then conducted a three-way ANOVA using the three stimulus factors on the aligned ranks. There were significant main effects of force [$df=2$, $F=49.0$, $p<0.001$, $\eta ^{2}_{p}=0.73$], and material compliance [$df=2$, $F=145.5$, $p<0.001$, $\eta ^{2}_{p}=0.89$]. There was no main effect of imitated shaking [$df=1$, $F=0.89$, $p=0.35$, $\eta ^{2}_{p}=0.02$]. The results of post-hoc test are shown in Supplementary Note 2. To summarize, the visual indentation depth increased with the applied force or material compliance. A comparison of effect size shows that the effect of material compliance was larger than that of force.

We hypothesized that the force rating scores would increase with the visual indentation depth.

### Brief description of experimental procedure

In this experiment, observers were asked to watch video clips in which a material was deformed by being pressed with the index finger of an actor’s right hand, and to report how much force the actor seemed to apply to the material on a 100-point visual analog scale. In addition to the force estimation, we explored how estimation of the softness of the material varied with the parameters we wanted to check. Previous studies have shown that the amount of deformation of a material strongly affects the perceived softness^22,23^. On the other hand, it is not clear how visual shaking could influence the estimation of softness. We explored how both imitated shaking and the amount of deformation of the material affected the apparent softness of the material. In addition, we explored how the force and softness estimations influenced each other. Half of the observers rated force first and softness second, after watching a video clip. The other half of the observers rated softness first and force second.

### Force rating

**Figure 3:**
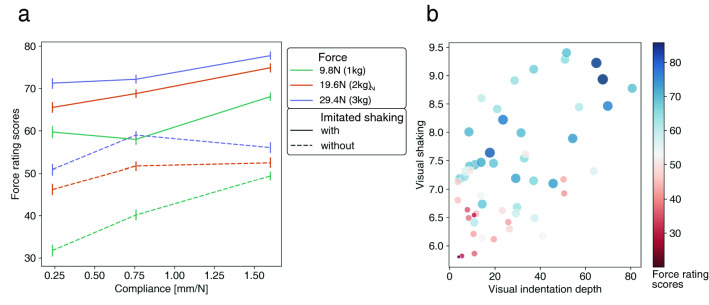
**(a)** Force rating scores for force, material compliance, and imitated shaking conditions. Error bars denote 95%CI. **(b)** Force rating scores for visual indentation depth and visual shaking.

The force rating scores for stimulus factors (force, material compliance, and imitated shaking conditions) are shown in Fig. 3a. Because the difference in the rating scores among the three actors was beyond the scope of our investigation, we averaged the scores among them. We conducted an ART on the force rating scores and then conducted a three-way ANOVA using the three stimulus factors on the aligned ranks. As a result, there were significant main effects of force [$df=2$, $F=697.5$, $p<0.001$, $\eta ^{2}_{p}=0.12$], material compliance [$df=2$, $F=273.6$, $p<0.001$, $\eta ^{2}_{p}=0.05$] and imitated shaking [$df=1$, $F=4791.0$, $p<0.001$, $\eta ^{2}_{p}=0.31$]. According to the effect size, the main effect of imitated shaking was more critical than those of other stimulus factors. Also, the main effect of material compliance was minor. There were significant interaction effects ($p<0.001$) but the effect sizes $\eta ^{2}_{p}$ were less than 0.01 and the effects were minor. See the result of the post-hoc test of main effects in Supplementary Note 3.

The force rating scores for visual indentation depth and visual shaking are shown in Fig. 3b. A GLM was used to analyze the effect of visual features in the video clips on the force rating scores. We fitted a model that regressed force rating scores with visual shaking and visual indentation depth as factors. The result of the likelihood ratio test showed significant main effects of visual shaking [$df=1, {\chi }^2=3501.3$, $p<0.001$] and visual indentation depth [$df=1, {\chi }^2=118.6$, $p<0.001$]. The interaction was also significant [$df=1, {\chi }^2=414.3$, $p<0.001$]. The standardized partial regression coefficient of the visual shaking was $0.23 \pm 0.004$ and that of the visual indentation depth was $-0.02 \pm 0.004$. The comparison of standardized partial regression coefficients shows that the main effect of visual indentation depth was minor and that human observers mainly use visual shaking to estimate the force that the actor is applying to the material. Regarding the goodness of the fit of the GLM, Nagelkerke’s pseudo R-squared was 0.25.

### Softness rating

**Figure 4:**
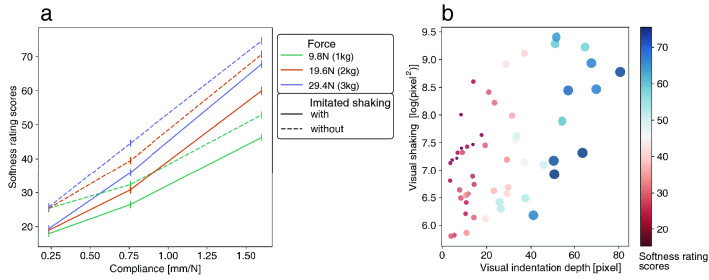
**(a)** Softness rating scores for force, material compliance, and imitated shaking conditions. Error bars denote 95%CI. (**b)** Softness rating scores for visual indentation depth and visual shaking.

The softness rating scores for each combination of force, material compliance, and imitated shaking conditions are shown in Fig. 4a. Because the difference in the rating scores among the three actors was beyond the scope of our investigation, we averaged the scores among them. We conducted an ART on the softness rating scores and then conducted a three-way ANOVA using the three stimulus factors on the aligned ranks. As a result, there were significant main effects of force [$df=2$, $F=483.4$, $p<0.001$, $\eta ^{2}_{p}=0.08$], material compliance [$df=2$, $F=6113.8$, $p<0.001$, $\eta ^{2}_{p}=0.53$] and imitated shaking [$df=1$, $F=672.5$, $p<0.001$, $\eta ^{2}_{p}=0.059$]. There were significant two-way interactions between force and compliance [$df=4$, $F=148.7$, $p<0.001$, $\eta ^{2}_{p}=0.052$], between force and imitated shaking [$df=2$, $F=3.5$, $p<0.001$, $\eta ^{2}_{p}=0.0006$], and three-way interactions [$df=4$, $F=3.4$, $p=0.009$, $\eta ^{2}_{p}=0.001$]. See the result of the post-hoc test of main effects in Supplementary Note 4.

The softness rating scores for visual indentation depth and visual shaking are shown in Fig. 4b. A GLM was used to analyze the effect of visual features in the video clips on the softness rating scores. We fitted a model that regressed softness rating with visual shaking and visual indentation depth as factors. The result of the likelihood ratio test showed significant main effects of visual shaking [$df=1, {\chi }^2=852.1$, $p<0.001$] and visual indentation depth [$df=1, {\chi }^2=268.8$, $p<0.001$]. The interaction between them was not significant [$df=1, {\chi }^2=0.1$, $p=0.72$]. The standardized partial regression coefficient of the visual shaking was $-0.18 \pm 0.006$ and that of the visual indentation depth was $0.52 \pm 0.006$. Though both of the two visual features had significant effects on softness rating, the absolute effect of the visual indentation depth was more critical than that of visual shaking. Regarding the goodness of fit of the GLM, Nagelkerke’s pseudo R-squared was 0.40.

To investigate how force and softness rating scores correlated with each other, we calculated Spearman’s rank correlation coefficient. The calculated coefficient was $\rho =0.06 (p=0.68)$, which showed there was no significant correlation between them.

## Discussion

The main purpose of the present study was to examine how human observers estimate, from visual information only, the force applied to a material by another person. We found that the force applied by another person was estimated to be larger with a greater magnitude of visual shaking in the actor’s finger and hand movement. The results add new evidence to the literature showing that human observers can visually read the kinematics of other people’s motor action^5–8^. It should be noted that the visual shaking varied not only with the applied force levels but also with the presence of imitated shaking applied by the actors in the video clips. Even when the force and material compliance factors in the video clips were constant, the force rating score changed depending on whether the imitated shaking was present or not (see Fig. 3a). This indicates that the visual shaking caused by imitated shaking can affect force estimation. We found that the human brain uses visual shaking of actors’ fingers and hands to estimate the level of force that is being applied. This is consistent with the prior findings about a mirror system related to the recognition of another person’s action^11,12^. The present study’s results indicate that the mirror system might work for the estimation of force applied by another person.

On the other hand, as compared to visual shaking, the visual indentation depth had a minor effect on the force rating. This is not consistent with our hypothesis. This may be because the observer attributed the variation in indentation depth to the material property rather than to the force applied by the actor. Indeed, the visual indentation depth affected the softness rating significantly, which was consistent with previous studies^22,23^.

Among the three stimulus factors we experimentally manipulated, force level and imitated shaking strongly affected the force rating, while the material compliance had only a minor effect on the force rating. The minor effect of material compliance on the force rating scores can be reasonably interpreted from the viewpoint of the two visual features. First, in respect of the visual indentation depth, as shown in Fig. 2c, the larger material compliance caused a larger visual indentation depth. Importantly, as shown in Fig. 3b and our GLM analysis, the visual indentation depth had a minor effect on the force rating scores. Given the weaker effect of the visual indentation depth on the force rating scores, it makes sense that the material compliance plays only a minor role in the determination of force estimation. Second, in respect of the visual shaking, although visual shaking strongly influenced the force rating scores, the effect of the material compliance on visual shaking was minor (Fig. 2c). That is, since the material compliance did not strongly modulate the visual shaking which was observable by the human visual system, the material compliance did not eventually affect the force rating scores too.

As stated above, there was a positive effect of material compliance on visual shaking. On the other hand, there was a negative effect of visual shaking on softness rating scores. The two results seem to be inconsistent with each other at first glance, but can be interpreted as follows. Instead of the observer estimating the softness directly from the visual shaking, the force applied by an actor might be firstly estimated on the basis of the visual shaking, and then, the softness was estimated through the consideration of the estimated force. That is, the observers might report a stiffer material when the material was pushed with “apparently” larger force than when it was pushed with “apparently” weaker force, since the visual indentation depth was almost constant despite the apparent strength of the applied force. Although this effect of visual shaking on softness estimation is intriguing, it should be noted that according to the standardized partial regression coefficients, the effect of visual shaking on the softness rating was minor compared to the effect of indentation depth on the softness rating.

**Figure 5:**
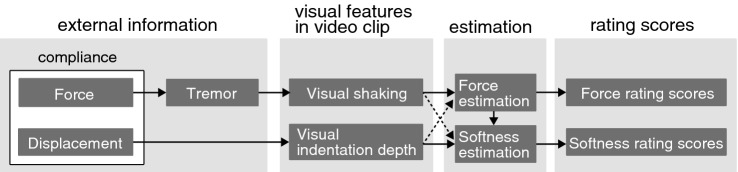
Flow of force and softness estimation from external information via visual features in video clip in our experiment.

Based on these discussions, we summarize an information flow from external information to the estimation of force and softness via visual features (see Fig. 5). Our results showed that the stimulus factors which we experimentally manipulated significantly contributed to the generation of visual features such as visual shaking and the visual indentation depth. In the figure, we organize the stimulus factors into “external information” consisting of the three components (force, displacement, and tremor). Here, we newly adopted a “displacement” because the displacement is required to understand the precise relationship among the force, the compliance, and the visual features. Please note that compliance is expressed by the displacement divided by the force. Hence, the external information in the figure contains the compliance as the combination of the displacement and the force. Our experimental results showed that both force levels and material compliance influenced the visual indentation depth. Importantly, however, there was no straightforward explanation about the relationship among them. We suggest that it is more straightforward to explain the relationship among them by adopting the displacement. In regard to the relationship between the force level and the visual indentation depth, as long as the material compliance is constant, as the force to indent an elastic material is larger, the magnitude of displacement increases, and this leads to the larger visual indentation depth in video clips. In regard to the relationship between the compliance and the visual indentation depth, as long as the force is constant, as the compliance is larger, the magnitude of displacement increases, and this likely leads to the larger visual indentation depth in video clips. In this way, the effects of the force levels or the material compliance on the visual indentation depth can consistently be explained with the involvement of the displacement. The later part of the flow in Fig. 5 shows how the visual features influence the estimation of force and softness, and also, how the estimations of force and softness interact with each other. The visual features independently affect the estimation of force and material softness. Based on the our results, there is a possibility that force estimation indirectly affects the softness estimation, which is indicated by the arrow from the force estimation to the softness estimation. Since we obtained no evidence that softness estimation affected the force estimation, the figure does not include the arrow from the softness estimation to the force estimation. In this way, our results summarized in Fig. 5 give novel insights into the structure of the mechanism for the estimation of force and material properties.

In this study, as stimuli, we used video clips in which an actor pushed the top surface of an elastic material with the index finger of his/her right hand, and found that the visual shaking is one of several effective cues for the estimation of the force being applied. Besides the situation we experimentally investigated, in everyday life there are many scenarios in which another person applies force to a material. For example, it is known that human observers can visually estimate the weight of a box that is lifted by another person^26^. It is an intriguing issue whether the estimate of weight and the lifter’s force can be influenced by visual shaking in the arm of the person lifting the box. Moreover, it is also an important issue whether the estimation of force applied by another person is influenced by characteristics of the person applying the force to the material. For example, future studies need to examine how age and gender inferred from the shape and size of an actor’s hand determine the visual estimation of force that the actor is applying. Finally, it is common to observe another person pushing or manipulating materials with tools such as knives, forks, chopsticks, scissors, baseball bats, shovels, etc. It is an interesting question whether the visual shaking of these tools also acts as a critical cue for the estimation of the force being applied.

## Methods

### Observers

In total, 600 people participated in the experiment as observers. The observers, with a mean age of 35.35 (SD: 8.51), consisted of three age groups (20s, 30s, and 40s) each made up of 100 men and 100 women. The 600 observers were classified into two groups each having 300 observers so that age and gender were balanced between the groups. Observers in one group answered with a softness rating first and a force rating second, after watching a video clip. Observers in the other group answered with a force rating first and a softness rating second. They were recruited online by a crowdsourcing research agent in Japan and were paid for their participation. Only people who could participate in the experiment using their own personal computers were recruited. They were unaware of the specific purpose of the experiment. Ethical approval for this study was obtained from the ethics committee at Nippon Telegraph and Telephone Corporation (Approval number: R02-009 by NTT Communication Science Laboratories Ethics Committee). The experiments were conducted according to the principles that have their origin in the Helsinki Declaration. Written informed consent was obtained from all observers in this study.

### Stimuli

The stimuli were video clips that showed an elastic cube, the top surface of which was pushed down by the index finger of an actor’s right hand. The video resolution was 288 $\times$ 512 pixels at 29.97 frames per second.

#### 3D printed material

We attempted to replicate three cubic metamaterials introduced in^27^ by using identical types of material (TangoBlackPlus) and 3D printer (Stratasys Obje500). Fig. 1a,c show photographs of the materials and their force–displacement curves respectively. The length of each edge of the cubes was 42 mm. Each cube contained 169 cylindrical holes. There were two sizes of hole for each material, which were different depending on the material as shown in Supplementary Table 2. The distance between the holes was 3.0 mm. These configurations were identical to those used in the previous study^27^. The cube’s surfaces in the previous study had a striped pattern artifact due to the cylindrical holes (see Fig. 1 in Piovarvci et al.’s paper^27^). If this striped pattern artifact is present on the surface of the cube, the surface texture differs depending on the configuration of the cylindrical holes. To remove the effect of any striped pattern artifact on the perception of softness, we made the surfaces flat.

To characterize the material property, we performed uniaxial load testing. An increasing force was applied to the cubes from the top so as to give a displacement at a constant speed of 1 mm / 6 seconds, and the corresponding force was recorded using a force tester (MCT-2150, A&D Co., Ltd.). Figure 1c shows the measured force–displacement curves. There was a difference in the force–displacement curve from the earlier study^27^. We consider that this could be attributable to the aforementioned absence of any surface striped pattern artifact in our materials. There is a possibility that the presence of striped pattern artifacts may have had an unintended effect on the force–displacement curves in the previous study^27^.

#### Video clip

We set the orientation of the material so that the surface with cylindrical holes was directed toward the actor. We filmed the videos from a position diagonally above the cube, so that the top surface pushed by the finger could be clearly seen. To rule out the effect of the holes on the force and softness estimations, we used image editing techniques to inpaint the holes with the color of the surrounding material (see Fig. 1b). The viewpoint of the camera was a position 30 cm in front of the material, 15 cm to the side, and 20 cm above. The camera lens was oriented towards the material. We filmed the actor’s right index finger pressing on the top of the material to deform it, and then relaxing his finger to return the material to its original shape. The actor wore a white glove to rule out any involvement of skin color change as a cue to force. The recorded scene in the video started from the time when the finger was stationary and in contact with the top surface of the material. Actors pushed the top surface of the material with three different levels of force, 1 kg (9.8 N), 2 kg (19.6 N), and 3 kg (29.4 N). When we video-shooted the material being pushed by the actors, the material was positioned on the digital weight scale (A&D SH12KN-JA). The actors could comprehend the force that they were applying to the material by reading out weight values at the display of the weight scale, and based on the displayed weight values, adjust the max forces. As the max force applied to the material, we extracted the maximum value in a sequence of displayed weight values in the raw video clip. See the actual max force applied for each material in Supplementary Fig. 1. To generate the video clips for stimuli, we cropped the video to the resolution of 288 $\times$ 512 pixels so that the weight scale display could not be seen. We shot videos for each material under the following two imitated shaking conditions: without imitated shaking, in which the actor pushed natparticipants were asked to lift upwards a white object initially at the ground line by dragging it with the computer mouse urally, and with imitated shaking, in which the actor shook his finger and hand while pushing. Between the two conditions, the main difference in visual signals was the presence of visual shaking due to the imitation of an induced tremor. There were three different actors. Thus, there were in total 54 video clips (3 actors $\times$ 3 material compliances $\times$ 3 force levels $\times$ 2 imitated shaking conditions). See Supplementary Videos corresponding to the 54 video clips and Supplementary Fig. 2 that shows snapshots of the stimulus videos without imitated shaking at 3 kg (29.4 N) force level as performed by one of the actors. The mean and standard deviation of the duration of 54 video clips was 6.65 ± 2.05 s. This deviation is attributed to the fact that we did not give actors strict instruction about how long the actors should press the material.

#### Quantifying visual features

To quantify the image information relevant to the indentation depth and the shaking, we computed optical flows using the Lucas-Kanade algorithm^28^ at the four image positions including the 1st joint, the 2nd joint, the 3rd joint, and the fingertip of the index finger of the actor. Regarding the indentation depth, the maximum amount of vertical displacement from the initial position of the fingertip was calculated as the visual indentation depth. The calculated visual indentation depth for each video clip is shown as the x-coordinate of each marker in Fig. 2b. In respect of the visual shaking, we conducted a fast Fourier transform for the temporal variations of the optical flow vectors along both horizontal and vertical dimensions at the four positions and obtained the logarithmic powers of amplitude spectra, which were summed across the horizontal and vertical dimensions as well as within each octave band (2–3 Hz, 4–7 Hz, and 8–15 Hz). To determine which octave band characterized the imitated shaking, we conducted an Aligned Rank Transform (ART)^25^ on the difference in logarithmic powers between the with and without imitated shaking conditions for each octave band. We conducted a two-way ANOVA using factors of the octave band on the aligned ranks. The main effect of the octave band was significant[$df=2, F=8.713$, $p<0.001$]. The post-hoc test revealed that there were significant differences between octave bands of 2–3 Hz and 4–7 Hz and between octave bands of 4–7 Hz and 8–15 Hz ($p<0.001$). This clarified the evident feature of visual shaking present in the 4–7 Hz octave band. Therefore, in this study, we used the logarithmic powers of the amplitude spectra of the optical flow vectors for the 4–7 Hz octave band as the feature of visual shaking for further analysis. The logarithmic power of the amplitude spectra for each material is shown as the y coordinate of each marker in Fig. 2b.

### Procedure

The experiment was programmed using jsPsych^29^. Each observer participated in the experiment in his or her own environment using a personal computer. Neither observation distances nor screen sizes were controlled. Although the presentation accuracy was not measured, it has been reported that stimulus timing control with jsPsych is sufficient to conduct online psychological experiments^30^.

After watching each video, observers estimated the applied force and softness and responded using a visual analog scale (VAS)^31^ ranging from 0 to 100 with 100 scale divisions. In the case of estimating force, the “No force at all” anchor was placed on the left side of the scale and the “Largest force that you can imagine” anchor was placed on the right side of the scale. In the case of answering softness, the “Not soft at all” anchor was placed on the left side of the scale and the “Softer than anything you can imagine” anchor was placed on the right side of the scale. The experiment was composed of an initial familiarization phase and then the actual test phases. In the familiarization phase, each observer was asked to provide answers for 3 randomly extracted video clips out of the 54 total video clips. After these were completed, the test phase started, in which each observer provided an answer for each of the 54 video clips. The presentation order of the 54 video clips was assigned pseudo-randomly to each observer.

### Data analysis

We investigated whether the rating scores for force or softness for the videos differed depending on the applied force level, material compliance, and the presence or absence of imitated shaking. For each observer and for each combination of the three factors, we calculated the mean rating scores using three rating scores corresponding to three actors. A three-way ANOVA was used for analysis. If there was a violation of normality check by the Shapiro-Wilk test, we conducted an ART on the data and then conducted the ANOVA on the aligned ranks. Since other conventional nonparametric statistical tests (e.g., Kruskal–Wallis test and Mann-Whitney U test) cannot test the effect of multiple factors and their interaction, we adopted the ART procedure (see details in the paper on ART^25^).

Next, a GLM was used to analyze the effect of visual indentation depth and visual shaking. Since the force rating is a positive continuous value, it was modeled by a logarithmic link function with a gamma distribution. To determine which factor was significant, a likelihood ratio test (Type II test) was performed. To determine which factor was more significant, standardized partial regression coefficients were compared. Nagelkerke’s pseudo R-squared was calculated to check how the GLM could explain our results.

## Supplementary Information

Supplementary Information 1.

Supplementary Information 2.

## Data Availability

The authors confirm that the data supporting the findings of this study are available within the article and its supplementary materials.

## Code availability

The code used for the analysis in this study is available from the corresponding authors upon request.
