# Supplementary material for: Visual estimation of the force applied by another person
Source: Sci Rep. 2022 Apr 13;12:6216. doi: 10.1038/s41598-022-10243-7 (PMC9008024; doi:10.1038/s41598-022-10243-7)
Supplement: Supplementary file 2 — Supplementary Information 2. [file 41598_2022_10243_MOESM2_ESM.pdf]

**Supplementary Information for**
**"Visual estimation of the force applied by another person"**

**Yusuke Ujitoko[1], Takahiro Kawabe[1]**
**[1]NTT Communication Science Laboratories**

**Correspondence:** Yusuke Ujitoko yusuke.ujitoko@gmail.com

**Contents:**

Supplementary Notes 1-4
Supplementary Figures 1-2
Supplementary Tables 1-2
Supplementary Videos 1-54

## Supplementary Notes

### Supplementary Note 1: Post-hoc tests on the main effects of stimuli factors on visual shaking.

As a result of post-hoc test of the main effect of force levels, there were significant differences in visual shaking between pairs of 1kg (9.8N)-2kg (19.6N) and 1kg (9.8N)-3kg (28.4N) ($p<0.01$), but there was no significant difference between pairs of 2kg (19.6N)-3kg (28.4N) ($p>0.05$).
As a result of post-hoc test of the main effect of material compliance, there were significant differences in visual shaking between pairs of High compliance-Low compliance, and High compliance and Middle compliance ($p<0.05$), but there was no significant difference between pairs of Low compliance and Middle compliance ($p>0.05$).

### Supplementary Note 2: Post-hoc tests on the main effects of stimuli factors on visual indentation depth.

As a result of post-hoc test of the main effect of force levels, there were significant differences in visual shaking between all pairs of force levels ($p<0.01$). Also, as a result of post-hoc testing of the main effect of material compliance, there were significant differences in visual shaking between all pairs of material compliance ($p<0.01$).

### Supplementary Note 3: Post-hoc tests on the main effects of stimuli factors on force rating scores.

As a result of post-hoc test of the main effect of force levels, there were significant differences in force rating scores between all pairs of force levels ($p<0.01$). Also, as a result of post-hoc test of the main effect of material compliance, there were significant differences in force rating scores between all pairs of material compliance ($p<0.01$).

### Supplementary Note 4: Post-hoc tests on the main effect of stimuli factors on the softness rating scores.

As a result of post-hoc test of the main effect of force levels, there were significant differences in softness rating scores between all pairs of force levels ($p<0.01$). Also, as a result of post-hoc test of the main effect of material compliance, there were significant differences in softness rating scores between all pairs of material compliance ($p<0.01$).

## Supplementary Figures

### Supplementary Figure 1: Maximum applied force by actor.

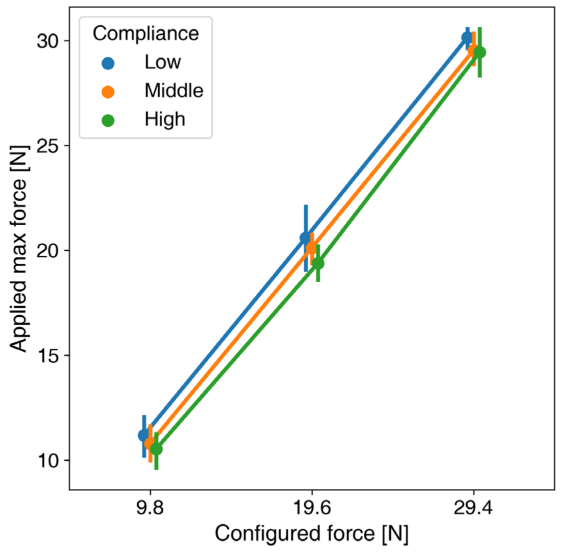


### Supplementary Figure 2: Snapshots of video without imitated shaking when the applied force is 3 kg (29.4 N).

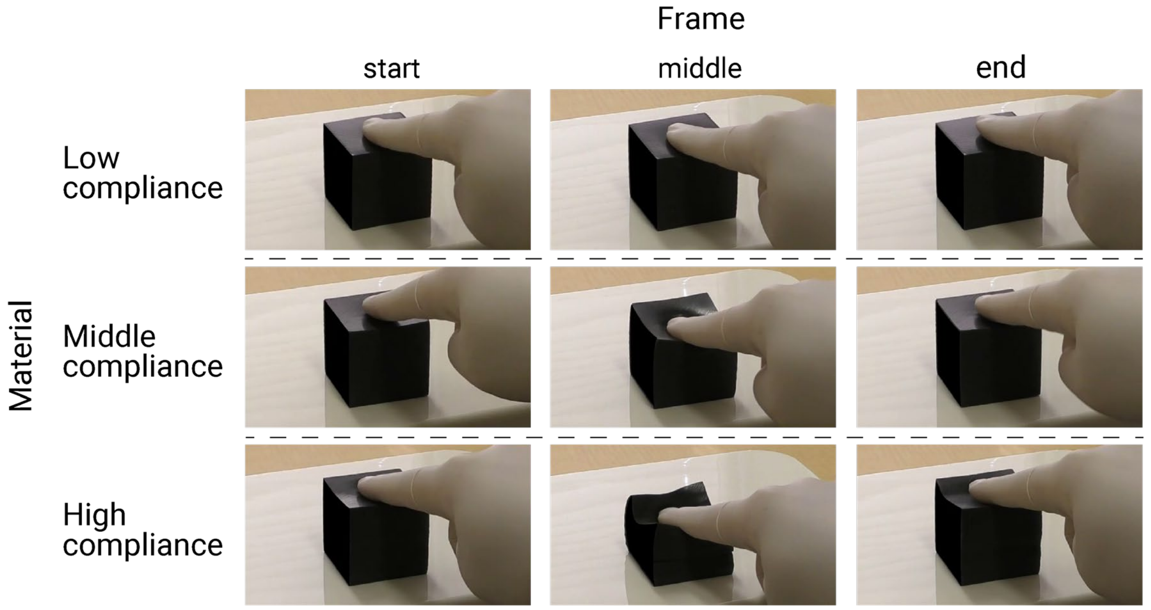


The starting, middle, and ending frames are shown in each row corresponding to each object.

## Supplementary Tables

### Supplementary Table 1: R squared value of linear models for each material.

| Material | R squared value |
|---|---|
| Low compliance | 0.917 |
| Middle compliance | 0.986 |
| High compliance | 0.993 |

Each column shows the diameter of the holes in each object.

### Supplementary Table 2: Diameter of the holes in the objects

| Material | Diameter of first size of hole[mm] | Diameter of second size of hole[mm] |
|---|---|---|
| Low compliance | 0.6 | 0.5 |
| Middle compliance | 1.4 | 0.95 |
| High compliance | 1.75 | 0.8 |

Each column shows the diameter of the holes in each object.

## Supplementary Videos

Supplementary videos 1-54 correspond to each combination of actor, compliance level, force level, and imitated shaking conditions. The naming rule of the filename is “[Actor number]_[Compliance level]_[Force level]_[Imitated shaking condition].mp4”. For example, the filename “Actor1_HighCompliance_Force1kg_WithoutShaking.mp4” means that actor number 1 pressed the high compliance material with 1kg (9.8N) of force without any imitated shaking.
